# Root endophytic *Aquapteridospora rhizicola* enhances growth and heavy metal tolerance of *Miscanthus sinensis* Andersson at a sedimentary site via indole-3-acetic acid and melanin production enhanced by high Fe and high temperature

**DOI:** 10.1371/journal.pone.0359285

**Published:** 2026-10-27

**Authors:** Yuta Matsushiro, Kohei Doyama, Toshikatsu Haruma, Xiang Lu, Hayato Masuya, Kei Shingai, Shingo Tomiyama, Hiroko Isoda, Keiko Yamaji

**Affiliations:** 1 Graduate School of Life and Environmental Sciences, University of Tsukuba, Tsukuba, Ibaraki, Japan; 2 Department of Mushroom Science and Forest Microbiology, Forestry and Forest Products Research Institute, Tsukuba, Ibaraki, Japan; 3 Mitsubishi Materials Corporation, Chiyoda, Tokyo, Japan; 4 Division of Sustainable Resources Engineering, Faculty of Engineering, Hokkaido University, Sapporo, Hokkaido, Japan; Universitat Jaume 1, SPAIN

## Abstract

*Aquapteridospora rhizicola*, a dark septate endophyte, was isolated from the roots of *Miscanthus sinensis* Andersson, which grows naturally as pioneer grass at a sedimentary site in an old mine. In the early successional stage, *M. sinensis* seedlings begin to grow on bare ground under heavy metal stress (especially Fe) and sharp soil temperature fluctuations. This study aimed to clarify the effects of root-endophytic *A. rhizicola* on *M. sinensis* seedling growth and tolerance, in bare-ground soil, while considering the sharp alteration in soil temperature of the bare ground at the study site. We conducted inoculation tests using sterilized bare soil under normal and actual soil temperature conditions, thereby mimicking the bare ground at the study site. Although *A. rhizicola* did not promote seedling growth under normal temperature conditions, this fungus enhanced plant growth under actual soil temperature conditions. To elucidate the underlying mechanism of these results, we analyzed the production of indole-3-acetic acid (IAA) and melanin by *A. rhizicola* under high Fe and high temperature conditions. The root endophyte increased IAA production in response to the combined conditions of high Fe and high temperature, and this might have contributed to the growth of *M. sinensis*. Moreover, *A. rhizicola* might alleviate heavy metal toxicity in the host plant by suppressing the translocation of toxic Zn from roots to terrestrial parts under actual soil temperature conditions. This might be explained by the high production of melanin, which possesses heavy metal binding capacity and antioxidant activity, and whose effect was enhanced under conditions of high Fe and high temperature. Our results indicate that *A. rhizicola* might enhance growth and heavy metal tolerance of *M. sinensis* in bare ground of the study site, via increased IAA and melanin production by habitat environmental factors, such as high Fe and high temperature.

## Introduction

Proper maintenance of metalliferous mine waste following the cessation of mining activities is essential to protect the surrounding ecosystems and health of local residents. Japan has over 5000 closed or abandoned metal mines, with approximately 100 of these sites producing mine drainage that is often acidic [1]. Its low pH coupled with the presence of heavy metals makes acidic mine drainage one of the most significant environmental challenges faced by the mining industry worldwide [2,3]. Acidic mine drainage can be neutralized using lime etc., which results in the production of sludge containing high concentrations of heavy metals [4]. This sludge is stored at a sedimentary site after being dewatered using a press filter. Many countries have made rehabilitation of mine sites, including sedimentary sites, mandatory by legal frameworks [5–7]. Notably, vegetation plays an important role in rehabilitation by providing the following functions: 1) reduction of surface erosion caused by wind and water, 2) reduction of percolating water through transpiration, and 3) improvement of landscape [8–10].

Natural vegetation has been observed at mine sites, thereby suggesting that plants naturally growing in sedimentary sites have evolved diverse heavy metal tolerances to adapt to heavy metal-enriched environments [11]. The poaceous pioneer perennial grass, *Miscanthus sinensis* Andersson, grows at several mine sites in Eastern Asia, including Japan [12,13], China [14], and Korea [15]. Several studies have reported that *M. sinensis* grows naturally at mine sites and accumulates high concentrations of metals such as Al [16], Pb, Zn [17,18], As, and Sb [19]. Our previous research revealed that *M. sinensis*, which grows at mining sites, has two major metal tolerance mechanisms: 1) exclusion of metals from living parts into dead parts, such as the root bark [18,20], and 2) production of detoxicants, which chelate with metals and capture reactive oxygen radicals generated under metal stress, such as chlorogenic acid [16,18], citric acid, and malic acid [16]. Our study site is a sedimentary site of a former mine in Japan, where *M. sinensis* grows naturally and forms patches (S1 Fig). Our previous research at this site discovered that Japanese red pine (*Pinus densiflora* Siebold & Zucc.) seedlings grew better inside *M. sinensis* patches than outside the patches. This indicates that *M. sinensis* facilitates the establishment of *P. densiflora* seedlings [20]; therefore, *M. sinensis* patches would be an important area for vegetation succession at the sedimentary site. In contrast, the outside of the patches is bare ground without vegetation, and the soil displays sharp increases in temperature compared with the inside of the patches [20]. During the early stage of establishment, *M. sinensis* seedlings should be established on bare ground under severe environmental conditions, such as heavy metal stress (especially Fe) and sharp fluctuations in soil temperature.

Root endophytes have been reported to enhance stress tolerance in host plants, including tolerance to heavy metals, heat, drought, salt, and pathogens [21]. Endophytes are defined as “fungi or bacteria which, for all or part of their life cycle, invade the tissues of living plants and cause unapparent and asymptomatic infections entirely within plant tissues but cause no symptoms of disease” [22]. Recent studies have shown that dark septate endophytes (DSEs), a class of root endophytes with melanized structures including inter- and intracellular hyphae and microsclerotia [21,23], induce plant tolerance to heavy metal stress [18,24–28]. Specifically, DSEs enhance heavy metal tolerance in host plants via promotion of nutrient uptake [18,26,28], immobilization of heavy metals within the root system [17,24,25], and production of indole-3-acetic acid (IAA) [18,28,29]. Melanin possesses heavy metal-binding capacity [30,31] and antioxidant activity [32], thereby suggesting that melanin in DSEs can support plants in tolerating heavy metal stress. It has been demonstrated that DSEs isolated from *M. sinensis* enhance growth and metal tolerance of the host plant in mine soil [18,28]. In our previous study [20], *Aquapteridospora* sp*.* was isolated as a novel DSE from the roots of *M. sinensis* growing at our study site, and was identified as *Aquapteridospora rhizicola* [33]. Importantly, it remains unclear whether *A. rhizicola* can improve the adaptation of *M. sinensis* to severe environments in sedimentary sites, or not.

In this study, we focused on the early stage of *M. sinensis* establishment at the sedimentary site. *Miscanthus sinensis* seedlings should start growing on bare ground at our study site under heavy metal stress (especially Fe) and sharp fluctuations in soil temperature. Through the inoculation tests of *M. sinensis* using *A. rhizicola* in sterilized bare soil collected from the sedimentary site, this study aimed to clarify the effects of root-endophytic *A. rhizicola* on *M. sinensis* seedling growth and tolerance, while considering the sharp alteration in soil temperature of the bare ground at the study site. The following two inoculation tests were set: 1) normal temperature conditions, following previous inoculation tests using *M. sinensis* [16,18,28,34], and 2) actual soil temperature conditions to mimic the soil temperature of the bare ground at the sedimentary site. Following the inoculation tests, we measured seedling growth, including height, root length, fresh weight (FW), and dry weight (DW), and analyzed elemental concentrations, including metal translocation and bioaccumulation abilities, and phenolic production in *M. sinensis* seedlings to compare seedlings inoculated with or without *A. rhizicola*. Since inoculation results indicated that *A. rhizicola* might promote *M. sinensis* growth under actual soil temperature conditions, we analyzed IAA and melanin production by *A. rhizicola* under high Fe and high temperature conditions. Through these experiments, we discussed the effects of *A. rhizicola* on the growth and heavy metal tolerance of *M. sinensis* in the habitat environment, focusing on IAA and melanin produced by the root endophyte.

## Materials and methods

### Inoculation tests of *M. sinensis* seedlings with *A. rhizicola* under normal and actual soil temperature conditions

#### Sterilization of collected soil from the sedimentary site and its properties.

With the permission of the property-owning corporation, the fieldwork was conducted at an old mine site in Akita Prefecture, Japan. We did not collect any protected species in this study. In September 2019, soil (200 mm × 200 mm × 200 mm) was randomly collected from four outside *M. sinensis* patches at the sedimentary site [20]. Concentrations of Al, Cu, Fe, Mn, and Zn in the air-dried and sieved (< 2 mm) soil after digestion in concentrated HNO_3_–HClO_4_ (1:4 v/v) at 140°C were 52.9 ± 5.9, 10.7 ± 1.2, 165.5 ± 18.3, 1.5 ± 0.2, and 19.0 ± 2.0 g/kg DW, respectively, while pH (H_2_O) was 7.73 ± 0.02 [20]. This study used soil sterilized by 30 kGy γ-ray irradiation; therefore, pH (H_2_O), available P and Fe, and exchangeable Ca, K, Mg, and Na were analyzed to evaluate the effect of γ-ray sterilization on the soil chemical characteristics. pH (H_2_O) was determined using a pH meter (F-22; HORIBA, Kyoto, Japan; soil: water, 1:2.5 w/v). Available Fe was extracted according to a previously described method [35] as follows: 2 g of soil was added to 1 M sodium acetate solution (20 mL, pH 4.8), and available Fe was extracted by shaking at 100 rpm for 2 h. Available P was extracted from 0.5 g soil using 0.5 M sodium hydrogen carbonate (10 mL, pH 8.0) and activated carbon (0.1 g) by shaking the mixture at 100 rpm for 30 min [36]. Exchangeable Ca, K, Mg, and Na were extracted from the soil using 1 M ammonium acetate (pH 7.0) by shaking at 100 rpm for 1 h [36]. The concentrations of available Fe and P, and exchangeable cations (Ca, K, Mg, and Na) were measured using inductively coupled plasma optical emission spectrometry (ICP-OES; Optima 7300 V, PerkinElmer, Massachusetts, USA). For all measurements, the results of four replicates were averaged, and standard errors (SEs) were calculated (S1 Table).

On a clean bench, the sterilized soil (20 g) was transferred to a sterilized culture tube (120 mm height × 30 mm i.d.; AGC Techno Glass Co., Shizuoka, Japan) with a polypropylene cap (AGC Techno Glass Co.). Thereafter, sterile deionized water (10 mL) prepared by a Millipore filtration system (Elix® Essential 15 UV, Merck, Darmstadt, Germany) was added to each tube.

#### Preparation of sterile seedlings.

Seeds of *M. sinensis* were collected in the area around the study site in September 2020 and 2022, and kept at 4°C until use. The seeds collected in September 2020 were used for the inoculation test under normal temperature conditions, and the seeds collected in September 2022 were used for the inoculation test under actual soil temperature conditions (see section “Growth conditions under normal temperature and actual soil temperatures”).

The sterilization procedure for the seeds followed a modified version of a previously described method [16]. The seeds were soaked in deionized water for 5 min and surface-sterilized with 70% ethanol for 1 min, 7.5% hydrogen peroxide solution for 5 min, and 70% ethanol for 1 min. After rinsing with sterile deionized water, the seeds were incubated on 1/3 Hoagland medium containing 1.5% agar for three weeks (light: 14 h, 25°C/dark: 10 h, 20°C) (LH-120S, Nippon Medical & Chemical Instruments, Osaka, Japan). Seedlings at the second- or third-leaf stage were used for the inoculation tests.

#### Mycelial suspensions for the inoculation.

*Aquapteridospora rhizicola* (NBRC116724), frequently isolated from the roots of *M. sinensis* growing naturally at the study site and identified using DNA analysis [20,33], was used in this study. This root endophyte had dark hyphae and septate, and was considered a DSE. *Aquapteridospora rhizicola* was previously grown on 1% malt extract agar (1% MA) at 23°C in the dark for two weeks, and 15 mycelial disks (5.5 mm i.d.) on the edge of the mycelia were inoculated into a 300-mL Erlenmeyer flask containing 100 mL of 1% malt extract liquid medium. Ten flasks were statically incubated at 23°C in the dark for two weeks and then filtered through a sterile tea ball to obtain mycelia. The mycelia were rinsed with sterile deionized water to remove the medium and then homogenized in sterile deionized water using a homogenizer (T 25 digital ULTRA-TURRAX, IKA, Osaka, Japan). Sterile deionized water was added to prepare a mycelial solution containing 3.8 mg of mycelial DW in 500 µL.

#### Growth conditions under normal temperature and actual soil temperatures.

One sterile seedling was transplanted aseptically into each culture tube. The following two treatments were prepared: 1) seedlings inoculated with *A. rhizicola* mycelial solution (3.8 mg of mycelial DW in 500 µL was put around the roots), and 2) seedlings with 500 µL of sterile deionized water as the control. Additionally, the following two growth conditions were prepared: 1) normal temperature conditions (light: 14 h, 25°C/dark: 10 h, 20°C) in the growth chamber (LH-100S, Nippon Medical & Chemical Instruments Co., Osaka, Japan), following the previous inoculation tests using *M. sinensis* [16,18,28,34], and 2) actual soil temperature conditions (light: 14 h, 31°C/dark: 10 h, 21°C) to mimic the soil temperature outside *M. sinensis* patches (bare ground) at the study site. In our previous research [20], soil temperature was measured at a depth of 5 cm, where *M. sinensis* roots are most abundant, during the growing season from July to September 2022. We selected the week with the highest maximum soil temperature and the week with the lowest minimum soil temperature during the measurement period (maximum: 32.3 ± 0.3°C / minimum: 20.8 ± 0.2°C). The soil temperature at a depth of 3 cm in the culture tube was monitored (TR-52i, T&D Co., Matsumoto, Japan) during incubation, and the average soil temperature was shown in S2 Table. For 2) actual soil temperature conditions, the average soil temperatures in the light and the dark were measured as 31.8 ± 0.0°C and 21.2 ± 0.0°C, respectively. Five seedlings were used per replicate, and four replicates were prepared.

After 35 days, seedlings were washed with tap water followed by deionized water to remove soil particles. The following growth parameters of all seedlings (twenty seedlings per treatment) were measured in each treatment: number of leaves, maximum leaf length, seedling height, FW, DW of terrestrial parts, FW of roots, and water content of terrestrial parts. Additionally, two seedlings per replicate were randomly used to measure root DW and water content in each treatment (eight seedlings per treatment). One seedling per replicate was used to measure the root length in each treatment (four seedlings per treatment). For fungal re-isolation, infection observation, elemental analysis, and phenolic analysis, one seedling per replicate was used (four seedlings were used for each analysis in total).

In order to confirm whether the inoculation test was successful or not, *A. rhizicola* was re-isolated and trypan blue-stained roots were observed microscopically (CX23, Olympus, Tokyo, Japan) following previously described procedures [34]. For re-isolation, the roots were surface-sterilized with 70% ethanol for 1 min, 3.75% hydrogen peroxide solution for 5 min, and 70% ethanol for 1 min. Subsequently, the roots were rinsed with sterilized deionized water to remove the reagents and cut into approximately 10 mm pieces using a sterile scalpel. Root pieces were placed on 1% MA and incubated at 23°C in the dark for three weeks. The results of four replicates were averaged, and SEs were calculated.

#### Measurement of elemental concentrations in plant tissues, and the calculation of translocation and bioconcentration factors.

Terrestrial parts and roots were dried at 80°C for 48 h, ground, and pyrolyzed in concentrated HNO_3_ at 130°C. Nutrients (Ca, K, Mg, Na, P, and S) and metals (Al, Fe, Zn, Cu, and Mn) in plant tissues were measured using ICP-OES. The results of four replicates were averaged, and SEs were calculated.

The translocation factor (TF) can be used to calculate plant translocation capability and redistribution of metals throughout plant tissues, and the bioconcentration factor (BCF) can be used to estimate the ability of plants to accumulate metals from soil [37,38]. TF and BCF were calculated as follows [39]:


$$\begin{array}{c} \begin{array}{c} \text{TF}=\frac{M_{terrestrial\;parts}}{M_{roots}} \end{array} \end{array}$$ (1)

where *M*_*terrestral parts*_ was the metal concentration in the terrestrial parts, and *M*_*roots*_ was the metal concentration in the roots.


$$\begin{array}{c} \begin{array}{c} \text{BCF}=\frac{M_{terrestrial\;parts\;}or\;M_{roots}}{M_{soil}} \end{array} \end{array}$$ (2)

where, *M*_*soil*_ was the metal concentration in the soil. The results of four replicates were averaged, and SEs were calculated.

#### Analysis of phenolic compounds to detoxify metal toxicity.

The roots were cut into pieces with scissors in 100% methanol to extract phenolic compounds. The extract was filtered and evaporated *in vacuo* (REN-1000; Iwaki, Shizuoka, Japan). The concentrated sample was dissolved in 50% methanol (1 mL) and analyzed by high-performance liquid chromatography (HPLC; Prominence UFLC series, Shimadzu, Kyoto, Japan) using a Mightysil RP-18 GP column (75 × 4.6 mm i.d.; Kanto Chemical Co., Tokyo, Japan). Spectral characteristics were analyzed using a diode array detector (DAD; SPD-M20A, Shimadzu), according to a previously described method [40]. In order to identify the phenolic compounds in the root extracts, the spectral characteristics from 220 to 400 nm and retention times of chlorogenic acid (MP Biomedicals LLC., Santa Ana, CA, USA) were compared with those of the peaks in the root extracts. Following the methodology of Haruma et al. [20], high-performance liquid chromatography/electrospray ionization-mass spectrometer (HPLC/ESI-MS; LCMS-2020; Shimadzu) analysis was done under the following conditions: column, Mightysil RP-18 MS (150 × 2.0 mm i.d.; Kanto Chemical Co.) eluent, 0.1% formic acid (FUJIFILM Wako Pure Chemical, Osaka, Japan) in ultrapure water (FUJIFILM Wako) (solvent A) and 100% acetonitrile (FUJIFILM Wako) (solvent B); flow rate, 0.2 mL/min; injection volume, 10 µL; column temperature, 40°C. The following gradient was used for the eluent system: 0 min, 100% A; 0–30 min, 50% A and 50% B; and 30–60 min, 100% B. The mass spectra and retention times of the target peaks in the root extracts were compared with those of chlorogenic acid (MP Biomedicals, LLC). An absolute calibration curve for chlorogenic acid was prepared using HPLC-DAD at 320 nm to quantify phenolic compounds. The results of four replicates were averaged, and SEs were calculated.

### Analysis of IAA and melanin production by *A. rhizicola* under high Fe and high temperature, imitating bare ground at the sedimentary site

#### Analysis of IAA production.

In order to confirm the influence of high Fe and high temperature on fungal IAA production at the study site, Czapek liquid medium supplemented with 0.1% (w/v) L-tryptophan (FUJIFILM Wako) (pH 6.5) [41] was used. Iron was selected as the most abundant heavy metal in the soil and roots of *M. sinensis* at the study site [20]. The following four conditions were prepared: 1) Czapek liquid medium at 20°C, 2) Czapek liquid medium at 30°C, which was soil temperatures measured in daytime of bare ground from July to September 2022 [20], 3) Czapek liquid medium with Fe at 20°C, and 4) Czapek liquid medium with Fe at 30°C. Iron was added as FeSO_4_·7H_2_O to a final concentration of 125 ppm in the Czapek liquid medium and pH was adjusted to be 6.5 with 1 M HCl. 125 ppm of Fe was moderate stress on *A. rhizicola* for two-week incubation at 23°C (S3 Table).

Twenty mycelial disks (5.5 mm i.d.), cut from the margins of young colonies of *A. rhizicola* grown on 1% MA for three weeks, were inoculated into a 300-mL Erlenmeyer flask containing 100 mL Czapek liquid medium and incubated under each temperature condition in the dark for three weeks. Following incubation, the culture medium was filtered and the mycelia were rinsed three times with deionized water. The filter paper with mycelia was dried in an oven at 80°C for 4 h. Mycelial DW was calculated as the difference between the DW of the filter paper with and without mycelia. The IAA concentration in the filtrate was determined using the Salkowski reagent-based spectrophotometric method [42]. As a high concentration of Fe in the culture filtrate could enhance the colorimetric reaction of the Salkowski reagent [43], the concentrated culture filtrate was applied to a solid-phase extraction column (Strata C18-E, Phenomenex, Torrance, CA, USA) to remove Fe with deionized water. The fraction containing IAA, which was eluted with methanol, was concentrated and mixed with Salkowski reagent (100 mL of 35% perchloric acid and 2 mL of 0.5 M FeCl_3_ solution) in a ratio of 1:2. After incubating the sample with Salkowski’s reagent in the dark for 25 min, the absorbance was measured at 530 nm using a UV-Vis spectrometer (UV-2450, Shimadzu). The concentration of the IAA produced was calculated using a standard curve of IAA (FUJIFILM Wako). The results of three replicates were averaged, and SEs were calculated.

#### Analysis of melanin production.

Melanin production was determined using potato dextrose agar (PDA; Becton, Dickinson and Co., NJ, USA) supplemented with 2% agar (pH 5.0). The following four conditions were prepared for melanin production analysis: 1) PDA at 20°C, 2) PDA at 30°C, 3) PDA with 125 ppm of Fe at 20°C, and 4) PDA with 125 ppm of Fe at 30°C. Iron was added as FeSO_4_·7H_2_O to a final concentration of 125 ppm in PD medium and the medium pH was adjusted to be 5.0. A mycelial disk (5.5 mm i.d.), cut from the margins of young colonies of *A. rhizicola* grown on 1% MA for three weeks, was placed on each plate and incubated at each temperature in the dark for three weeks. Mycelial diameters at right angles were measured and averaged (n = 3).

Melanin was extracted and quantified from *A. rhizicola* colonies using a previously described method [44,45]. Four disks (4.0 mm i.d.) were cut from each three-week-old colony; if the mycelial diameter was more than 20 mm, 7.0 mm disks were cut. The disks were boiled in 5 mL of deionized water for 5 min at 100°C, and then centrifuged at 3500 rpm for 20 min. The mycelial pellet was washed with deionized water and centrifuged again. The pigment was extracted by autoclaving with 1 M NaOH (3 mL) for 20 min at 121°C. The alkaline pigment extract was acidified to pH 2 with concentrated HCl to precipitate melanin. The precipitate was washed thrice with deionized water and dried *in vacuo*. The dried pellets were dissolved in 1 M NaOH (1 mL) at 80°C for 2 h and centrifuged at 3500 rpm for 30 min. The supernatant was transferred to fresh tubes and the absorbance was measured at 405 nm using a UV-Vis spectrometer. Melanin content (µg/cm^2^ of disks) was determined using a standard melanin (Sigma-Aldrich Co. LLC, St. Louis, USA). The results of three replicates were averaged, and SEs were calculated.

### Statistical analysis

Statistical analyses were performed using the IBM SPSS Statistics software for Windows (ver. 29.0.2.0, IBM, Armonk, NY, USA). Differences in concentration of available P and Fe, exchangeable Ca, K, Mg, and Na, and pH (H_2_O) between non-sterilized and γ-ray sterilized soils were evaluated using a two-tailed unpaired Student’s t-test. Since the seeds used for the two inoculation tests were collected in different years, resulting in differences in the size and developmental stage of the *M. sinensis* seedlings at the time of inoculation, statistical analysis was conducted independently for each condition such as “normal conditions” or “actual soil temperature conditions.” Differences in soil temperature between conditions during the inoculation tests were evaluated using a two-tailed unpaired Student’s t-test. Under each condition (normal or actual soil temperature conditions), seedling growth variables, water content of terrestrial parts and roots, elemental concentrations (metals: Al, Fe, Zn, Cu, and Mn; nutrients: Ca, K, Mg, Na, P, and S), TFs, BCFs, and concentration of chlorogenic acid were evaluated using a two-tailed unpaired Student’s t-test. The water content of terrestrial parts and roots was statistically analyzed after arcsine transformation. Differences among conditions in the production of IAA and melanin, mycelial DW, and colony diameter were evaluated using a one-factor analysis of variance (ANOVA) with the Scheffé post-hoc test. Nutrient elements, such as Mg and P, in the inoculation tests were analyzed by the Smirnov–Grubbs outlier test, and the data identified as outliers were omitted. Differences were considered statistically significant at p < 0.05.

## Results

### Inoculation test under normal temperature conditions: Effects of *A. rhizicola* on *M. sinensis* seedling growth, concentrations of elements, TFs and BCFs, and phenolic production

Based on the results of fungal re-isolation, the inoculation test was considered to have been carried out without contamination because no microorganisms were isolated from the seedling roots in the control; *A. rhizicola* was re-isolated from the inoculated seedling roots. Moreover, microsclerotia and endophytic hyphae were observed in trypan blue-stained roots inoculated with *A. rhizicola* (S2a and b Fig). The infection rates of microsclerotia and endophytic hyphae were 9.7 ± 2.5% and 60.7 ± 3.4%, respectively. Lignitubers (S2 Figc) were also observed as lignin accumulation in the plant cell wall, as a response of plants to infection [46,47].

*Aquapteridospora rhizicola* did not increase the number of leaves, maximum leaf length, height, root length, FW and DW of terrestrial parts, FW and DW of roots, or water content of terrestrial parts and roots (p > 0.05) (Fig 1, Table 1). Metals (Al, Fe, Zn, Cu, and Mn) and nutrients (Ca, K, Mg, Na, P, and S) in terrestrial parts and roots are shown in Figs 2 and 3. *Aquapteridospora rhizicola* did not enhance the concentrations of metal and nutrient elements in terrestrial parts or roots (p > 0.05). Additionally, there were no significant differences in the TFs and BCFs between treatments (p > 0.05) (Tables 2 and 3).

**Table 1:** Seedling growth parameters after the inoculation test under normal temperature conditions.

Growth parameters	Control	Inoculation	P-value
Number of leaves	5.0 ± 0.1	4.7 ± 0.1	0.153
Maximum leaf length (cm)	10.7 ± 0.8	10.0 ± 0.6	0.512
Height (mm)	69.2 ± 4.0	72.0 ± 4.3	0.637
Root length (cm)	52.0 ± 6.9	53.8 ± 7.2	0.864
Terrestrial part FW (mg)	25.5 ± 2.4	25.4 ± 2.0	0.986
Root FW (mg)	22.7 ± 2.6	22.1 ± 2.5	0.869
Terrestrial part DW (mg)	4.4 ± 0.4	3.9 ± 0.4	0.455
Root DW (mg)	2.6 ± 0.4	2.4 ± 0.3	0.707
Water content of terrestrial parts (%)	83.1 ± 0.6	84.9 ± 0.5	0.097
Water content of roots (%)	91.3 ± 0.3	91.1 ± 0.4	0.920

FW: fresh weight. DW: dry weight. There were no significant differences between the control and the inoculated plants using a two-tailed unpaired Student’s t-test (number of leaves, maximum leaf length, height, terrestrial part FW, root FW, terrestrial part DW, and water content of terrestrial part, n = 20; root DW and water content of roots, n = 8; and root length, n = 4). Means are shown with ± SE.

**Table 2:** TFs of metals in *M. sinensis* seedlings under normal temperature conditions.

Metals	Control	Inoculation	P-value
Al	0.21 ± 0.07	0.18 ± 0.10	0.794
Cu	0.04 ± 0.01	0.03 ± 0.01	0.691
Fe	0.05 ± 0.01	0.04 ± 0.01	0.687
Mn	0.36 ± 0.10	0.37 ± 0.10	0.940
Zn	0.20 ± 0.07	0.18 ± 0.01	0.794

There were no significant differences between the control and inoculated groups using a two-tailed unpaired Student’s t-test (p > 0.05, n = 4). Means are shown with ± SE.

**Table 3:** BCFs of metals in *M. sinensis* seedlings under normal temperature conditions.

(a) Terrestrial parts			
**Metals**	**Control**	**Inoculation**	**P-value**
Al	0.01 ± 0.00	0.01 ± 0.00	0.810
Cu	0.00 ± 0.00	0.00 ± 0.00	0.202
Fe	0.00 ± 0.00	0.00 ± 0.00	0.910
Mn	0.02 ± 0.00	0.02 ± 0.00	0.388
Zn	0.01 ± 0.00	0.01 ± 0.00	0.490
(b) Roots			
**Metals**	**Control**	**Inoculation**	**P-value**
Al	0.04 ± 0.01	0.07 ± 0.02	0.100
Cu	0.12 ± 0.01	0.09 ± 0.01	0.265
Fe	0.03 ± 0.01	0.05 ± 0.01	0.364
Mn	0.05 ± 0.01	0.06 ± 0.01	0.427
Zn	0.04 ± 0.00	0.05 ± 0.01	0.329

There were no significant differences between the control and inoculated groups using a two-tailed unpaired Student’s t-test (p > 0.05, n = 4). Means are shown with ± SE.

**Fig 1:**
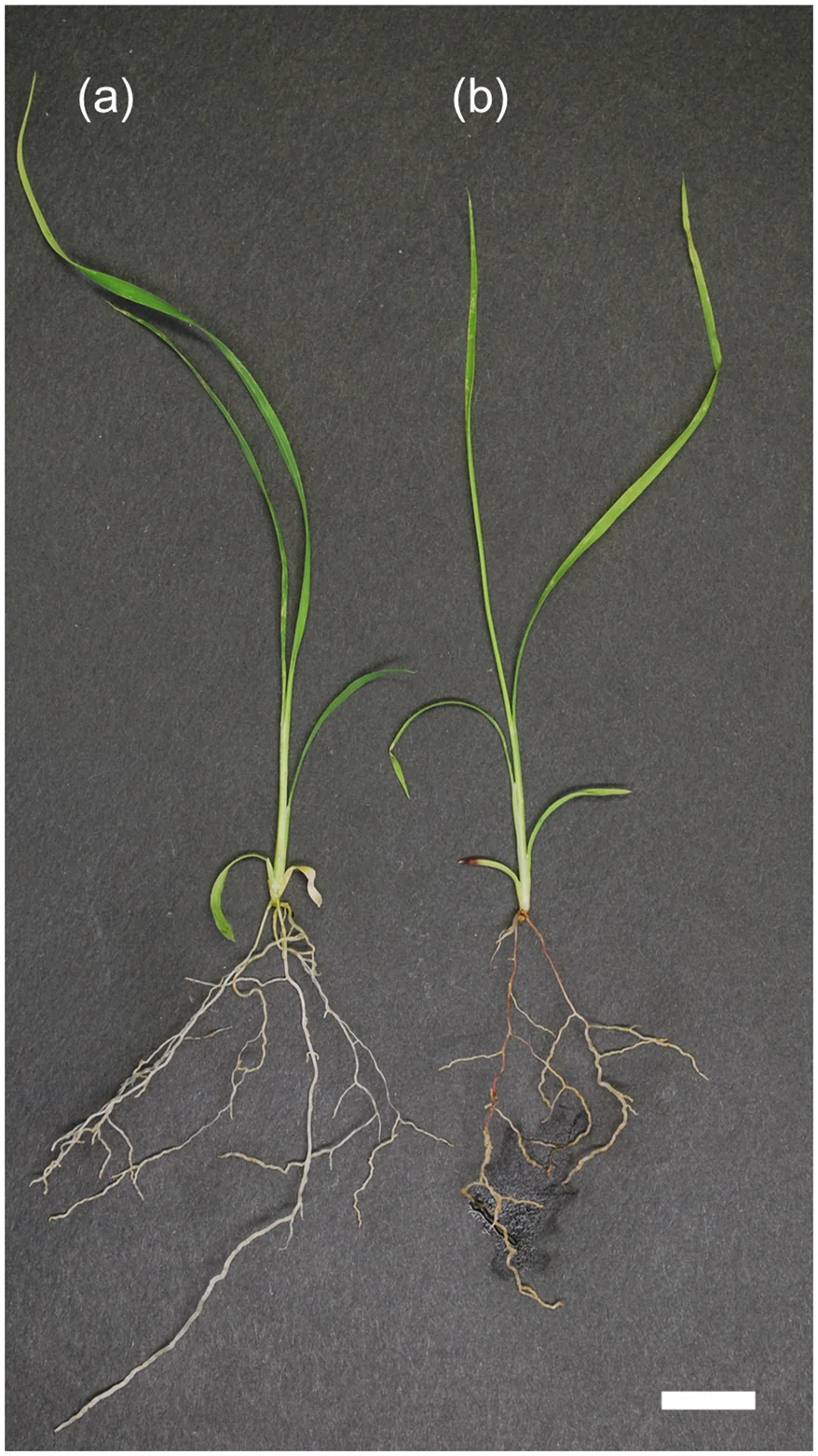
*Miscanthus sinensis* seedlings under normal temperature conditions. Seedlings were grown for 35 days in sterilized bare soil collected from the sedimentary site. (a) Control and (b) inoculation with *A. rhizicola.* Scale bar represents 10 mm.

**Fig 2:**
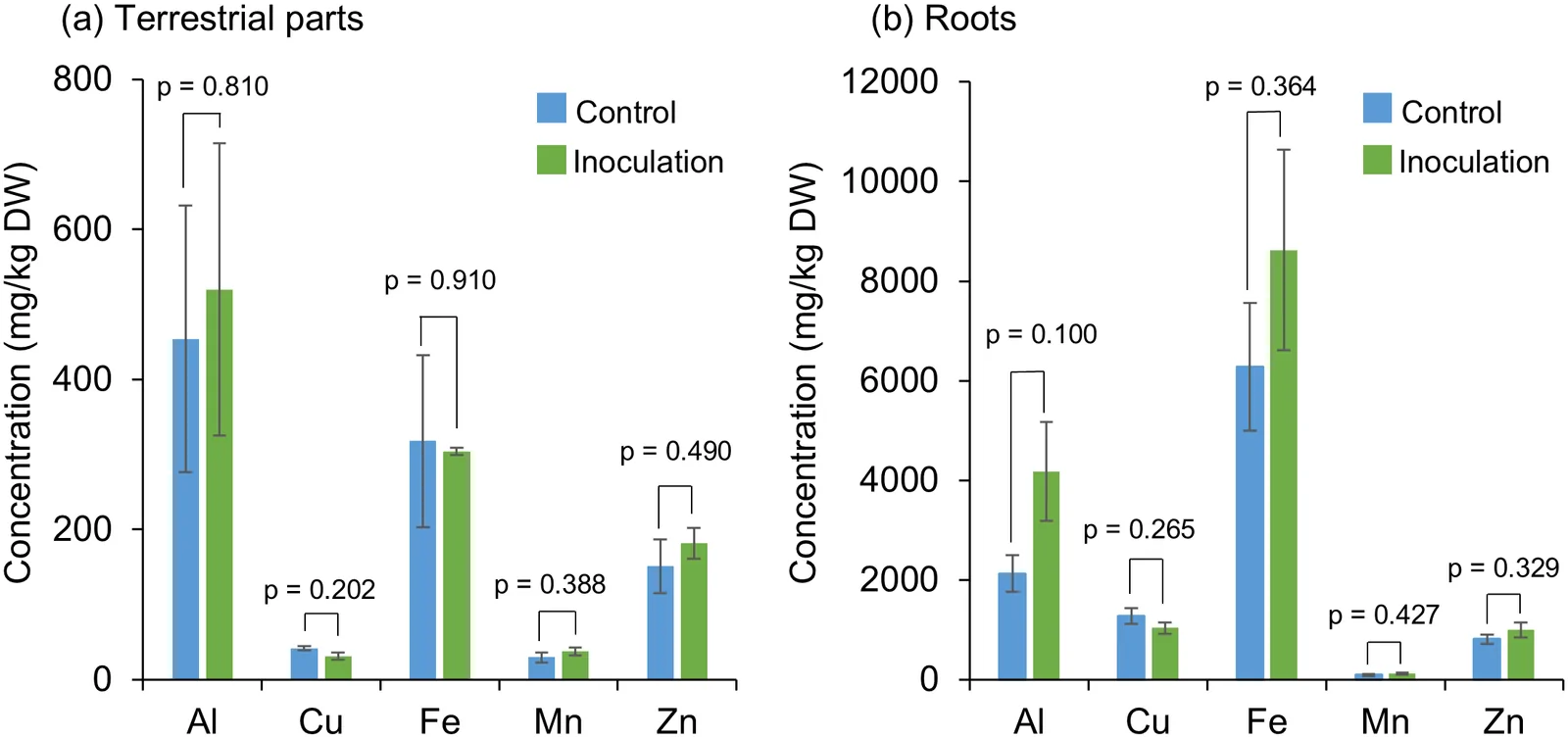
Metal concentrations in *M. sinensis* seedlings under normal temperature conditions. (a) Concentrations in terrestrial parts, and (b) concentrations in roots. There were no significant differences between the control and inoculated groups using a two-tailed unpaired Student’s t-test (p > 0.05, n = 4). DW: dry weight. Means are shown with ± SE.

**Fig 3:**
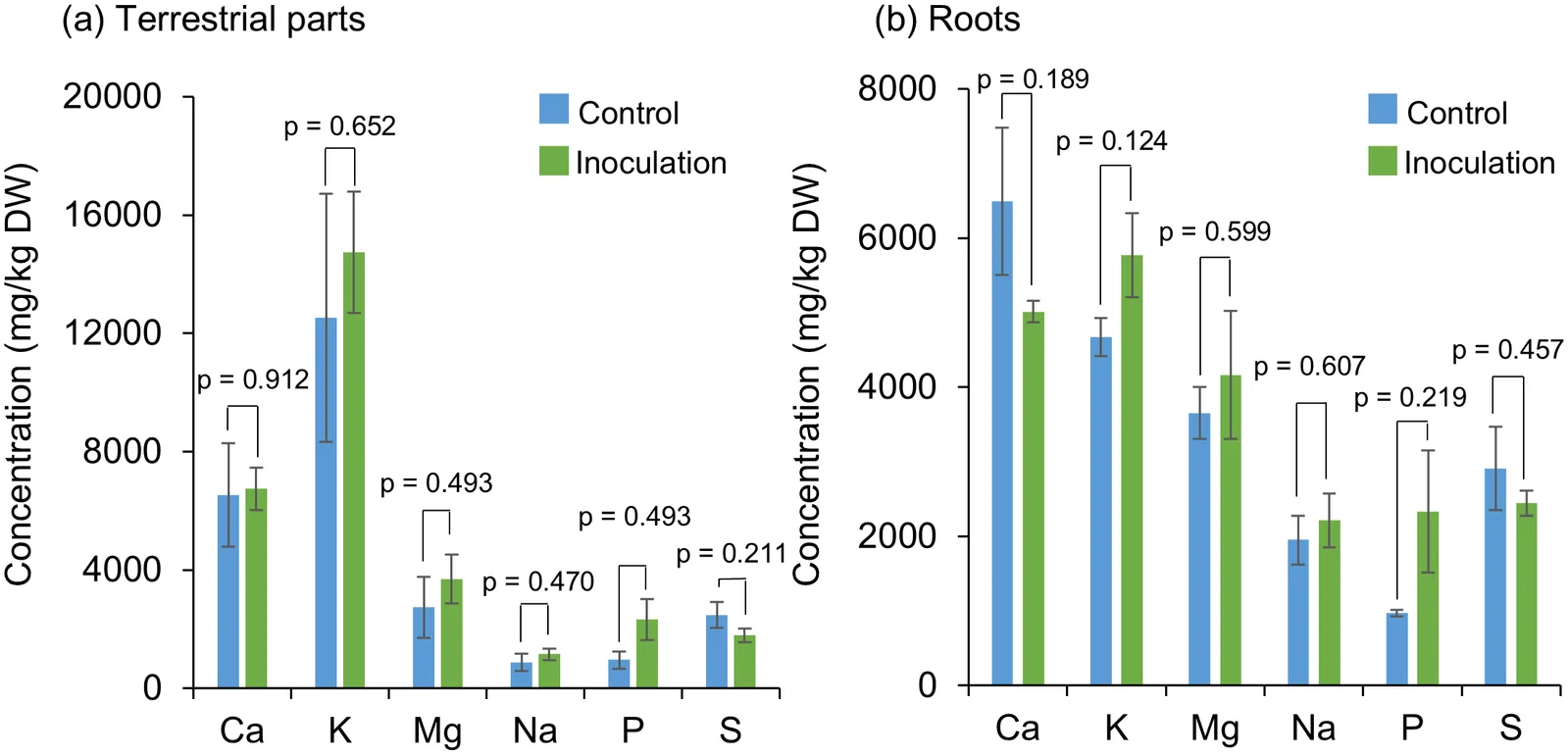
Nutrient element concentrations in *M. sinensis* seedlings under normal temperature conditions. (a) Concentrations in terrestrial parts, and (b) concentrations in roots. There were no significant differences between the control and inoculated groups using a two-tailed unpaired Student’s t-test (p > 0.05, n = 4). For P concentration in the terrestrial parts of the control, a datum was omitted by Smirnov–Grubbs test (p < 0.05, n = 3). DW: dry weight. Means are shown with ± SE.

The main phenolic compound in the root extract was chlorogenic acid. The concentrations of chlorogenic acid in control seedlings and *A. rhizicola*-inoculated seedlings were 0.01 ± 0.00 and 0.01 ± 0.00 µg/mg FW, respectively (n = 4). There were no significant differences in the concentration of chlorogenic acid between the treatments (two-tailed unpaired Student’s t-test, p = 0.608).

### Inoculation test under actual soil temperature conditions: Effects of *A. rhizicola* on *M. sinensis* seedling growth, concentrations of elements, TFs and BCFs, and phenolic production

The inoculation test was successful without contamination, as indicated by the re-isolation of *A. rhizicola* from the inoculated roots and not from the control roots. In trypan blue-stained roots inoculated with *A. rhizicola*, microsclerotia and endophytic hyphae (S2 Fig d and e) were confirmed, and the infection rates of microsclerotia and endophytic hyphae were 6.8 ± 2.5% and 19.3 ± 4.7%, respectively. Lignitubers (S2 Fig f) were also observed as lignin accumulation in the plant cell wall, as a response of plants to infection [46,47].

*Aquapteridospora rhizicola* enhanced *M. sinensis* seedling growth under actual soil temperature, thereby imitating the bare ground at the sedimentary site (Fig 4, Table 4). The maximum leaf length, FW of terrestrial parts and roots, height, and DW of terrestrial parts were significantly increased by inoculation of *A. rhizicola* (p < 0.05) (Table 4). *Aquapteridospora rhizicola* did not enhance the concentrations of metal elements in terrestrial parts or roots (p > 0.05) (Fig 5). In terrestrial parts, *A. rhizicola* significantly decreased Mg concentration (p = 0.005) (Fig 6a). In contrast, Mg concentration in roots was significantly higher in seedlings inoculated with *A. rhizicola* than in the control seedlings (p = 0.041) (Fig 6b). The TF of Zn in *A. rhizicola*-inoculated seedlings was significantly lower than that in the control seedlings (p = 0.048) (Table 5). There were no significant differences in the BCF values between the treatments (p > 0.05) (Table 6). Chlorogenic acid concentrations in control roots and *A. rhizicola*-inoculated roots were 0.06 ± 0.02 and 0.05 ± 0.01 µg/mg FW (n = 4), respectively. There were no significant differences in the concentration of chlorogenic acid among the treatments (two-tailed unpaired Student’s t-test, p = 0.834).

**Table 4:** Seedling growth parameters after the inoculation test under actual soil temperature conditions, imitating bare ground.

Growth parameters	Control	Inoculation	P-value
Number of leaves	5.1 ± 0.1	5.0 ± 0.2	0.824
Maximum leaf length (cm)	5.5 ± 0.4	6.8 ± 0.3**	0.008
Height (mm)	41.2 ± 2.6	51.1 ± 3.1*	0.019
Root length (cm)	26.6 ± 6.8	38.7 ± 6.5	0.245
Terrestrial part FW (mg)	20.5 ± 2.0	27.9 ± 1.9**	0.010
Root FW (mg)	14.1 ± 1.5	22.6 ± 2.3**	0.003
Terrestrial part DW (mg)	4.1 ± 0.5	5.8 ± 0.5*	0.012
Root DW (mg)	2.5 ± 0.2	2.8 ± 0.3	0.413
Water content of terrestrial parts (%)	80.6 ± 0.4	79.6 ± 0.4	0.134
Water content of roots (%)	86.5 ± 0.3	87.4 ± 0.2	0.178

FW: fresh weight. DW: dry weight. Differences between treatments were evaluated using a two-tailed unpaired Student’s t-test (* p < 0.05, ** p < 0.01; number of leaves, maximum leaf length, height, terrestrial part FW, root FW, terrestrial part DW, and water content of terrestrial parts, n = 20; root DW and water content of roots, n = 8; and root length, n = 4). Means are shown with ± SE.

**Table 5:** TFs of metals in *M. sinensis* seedlings under actual soil temperature conditions, imitating bare ground.

Metals	Control	Inoculation	P-value
Al	0.47 ± 0.12	0.30 ± 0.09	0.293
Cu	0.06 ± 0.01	0.07 ± 0.02	0.624
Fe	0.11 ± 0.04	0.08 ± 0.02	0.542
Zn	0.82 ± 0.04	0.53 ± 0.11*	0.048

Differences between treatments were evaluated using a two-tailed unpaired Student’s t-test (* p < 0.05, n = 4). The TFs of Mn could not be calculated because Mn concentrations in terrestrial parts and roots were below the detection limit. Means are shown with ± SE.

**Table 6:** BCFs of metals in *M. sinensis* seedlings under actual soil temperature conditions, imitating bare ground.

(a) Terrestrial parts			
**Metals**	**Control**	**Inoculation**	**P-value**
Al	0.02 ± 0.01	0.01 ± 0.01	0.575
Cu	0.00 ± 0.00	0.00 ± 0.00	0.785
Fe	0.00 ± 0.00	0.00 ± 0.00	0.076
Zn	0.08 ± 0.01	0.06 ± 0.00	0.127
**(b) Roots**			
**Metals**	**Control**	**Inoculation**	**P-value**
Al	0.04 ± 0.01	0.05 ± 0.01	0.773
Cu	0.07 ± 0.01	0.06 ± 0.01	0.434
Fe	0.03 ± 0.01	0.02 ± 0.00	0.540
Zn	0.10 ± 0.01	0.14 ± 0.04	0.423

There were no significant differences between the control and inoculated groups using a two-tailed unpaired Student’s t-test (p > 0.05, n = 4). The BCFs of Mn could not be calculated because Mn concentrations in terrestrial parts and roots were below the detection limit. Means are shown with ± SE.

**Fig 4:**
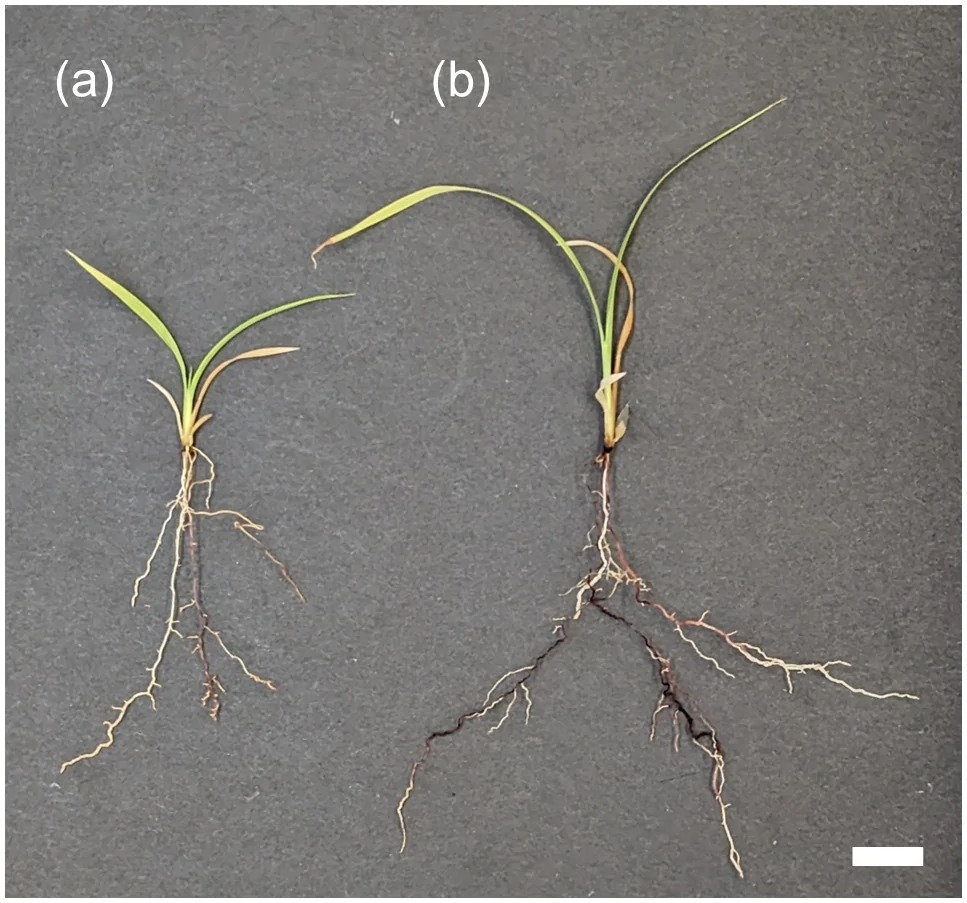
*Miscanthus sinensis* seedlings under actual soil temperature conditions, imitating bare ground. Seedlings were grown for 35 days in sterilized bare soil collected from the sedimentary site. (a) Control and (b) inoculation with *A. rhizicola.* Scale bar represents 10 mm. Roots in (b) are black, which could be attributed to *A. rhizicola* mycelia.

**Fig 5:**
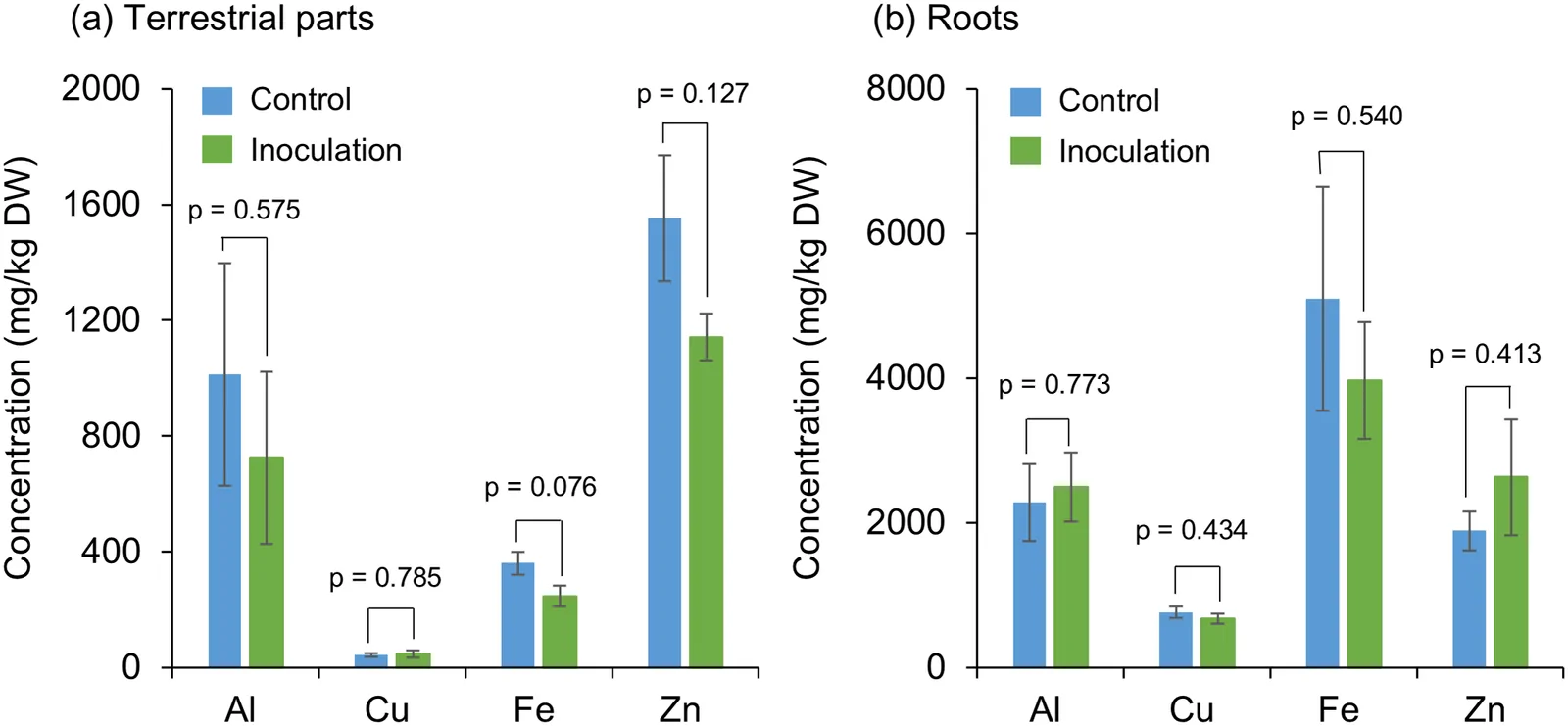
Metal concentrations in *M. sinensis* seedlings under actual soil temperature conditions, imitating bare ground. (a) Concentrations in terrestrial parts, and (b) concentrations in roots. There were no significant differences between the control and inoculated groups using a two-tailed unpaired Student’s t-test (p > 0.05, n = 4). DW: dry weight. Means are shown with ± SE. In our analysis, Mn concentrations in terrestrial parts and roots were below the detection limit.

**Fig 6:**
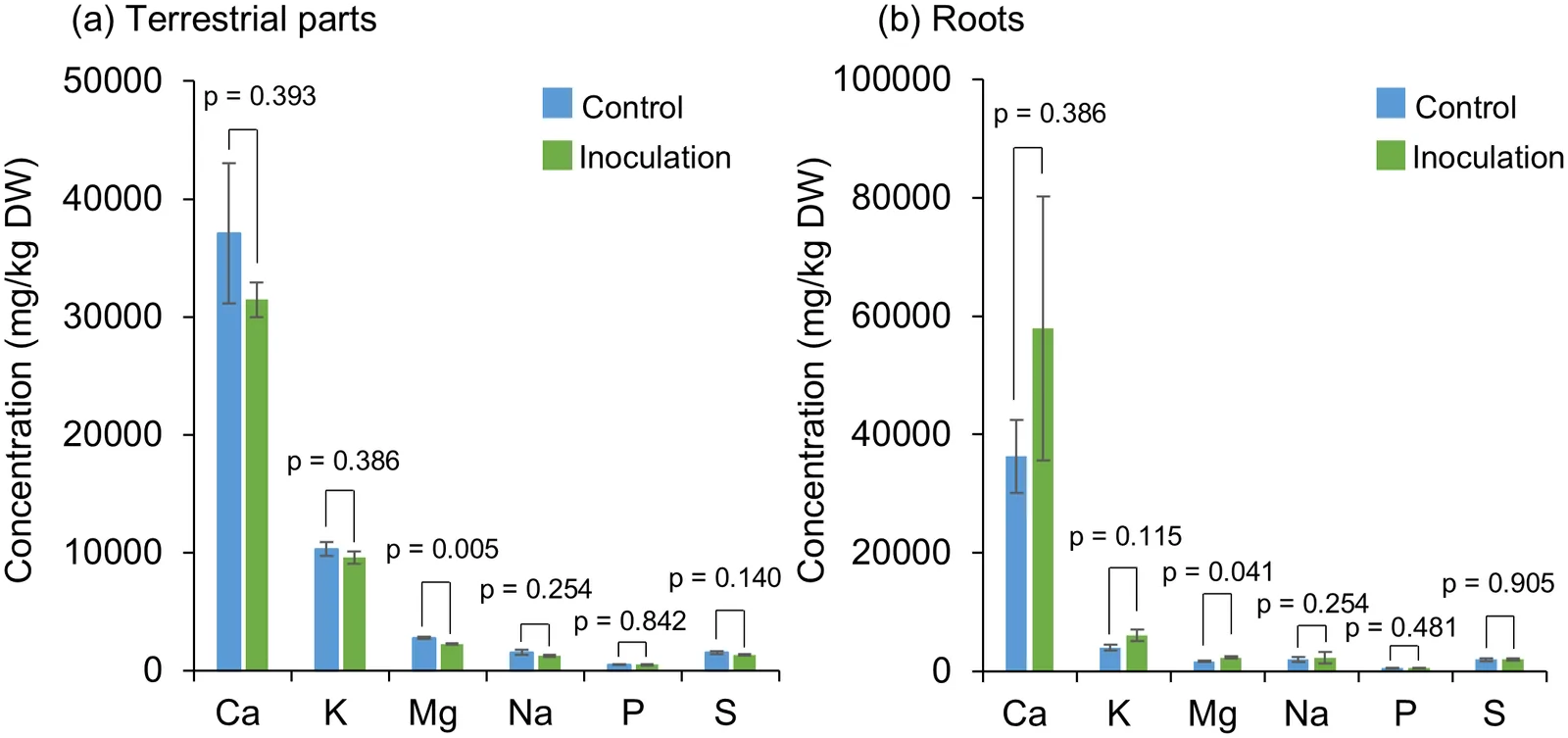
Nutrient element concentrations in *M. sinensis* seedlings under actual soil temperature conditions, imitating bare ground. (a) Concentrations in terrestrial parts, and (b) concentrations in roots. Differences between treatments were evaluated using a two-tailed unpaired Student’s t-test (n = 4). DW: dry weight. Means are shown with ± SE.

### IAA and melanin production by *A. rhizicola* under high Fe and high temperature, imitating bare ground at the sedimentary site

*Aquapteridospora rhizicola* was incubated under normal (20°C) /high temperature (30°C) and with/without high Fe to understand its response to bare ground at the sedimentary site. Mycelial DW was significantly reduced under high temperature (30°C) (p = 0.006), with Fe (20°C) (p = 0.012), and combined condition of high temperature (30°C) and high Fe (p < 0.001) compared with the control (20°C) (Fig 7a). Despite growth inhibition, IAA production per unit of mycelial DW significantly increased under the combined condition of high temperature and high Fe concentration (p < 0.001) (Fig 7b). Melanin production on PDA medium under the same conditions described above was also examined. Significant inhibition of fungal growth on PDA was confirmed at high temperature (p < 0.001), high Fe concentration (p = 0.003), and combined condition of high temperature and high Fe concentration (p < 0.001) compared with the control (Fig 8a). However, melanin production significantly increased at high temperature (p < 0.001), and combined condition of high temperature and Fe concentration (p = 0.020) (Fig 8b).

**Fig 7:**
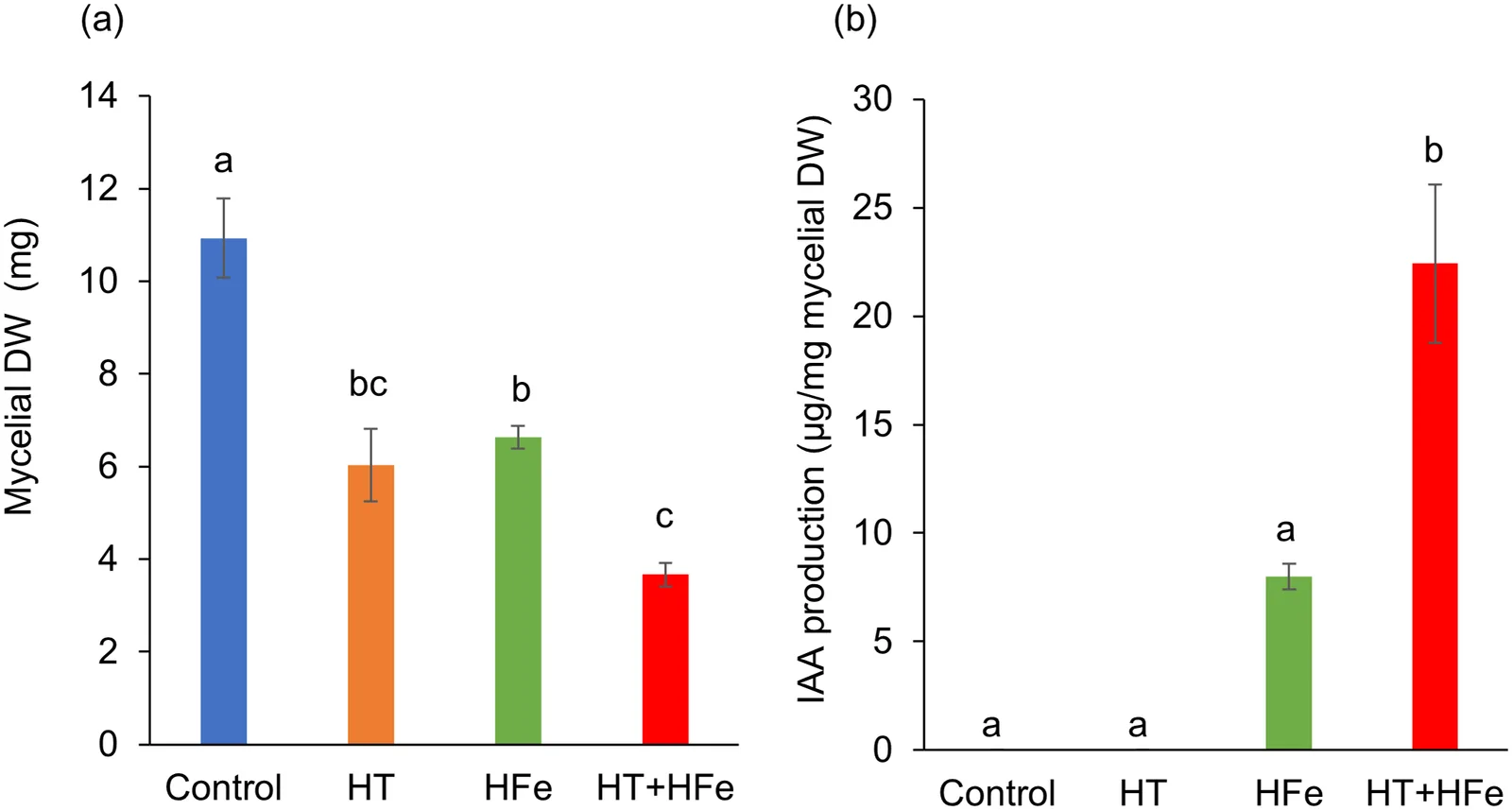
Mycelial DW and IAA production of *A. rhizicola* incubated under high Fe and high temperature. *Aquapteridospora rhizicola* was incubated in Czapek medium under control (20°C), high temperature (HT) (30°C), high Fe (HFe) (20°C), and high temperature and high Fe (HT + HFe) (30°C) for three weeks. (a) Mycelial DW of *A. rhizicola*, and (b) IAA production by *A. rhizicola*. Different letters indicate statistically significant differences among conditions in a one-factor ANOVA, Scheffé’s post-hoc test, p < 0.05 (n = 3). DW: dry weight. Means are shown with ± SE.

**Fig 8:**
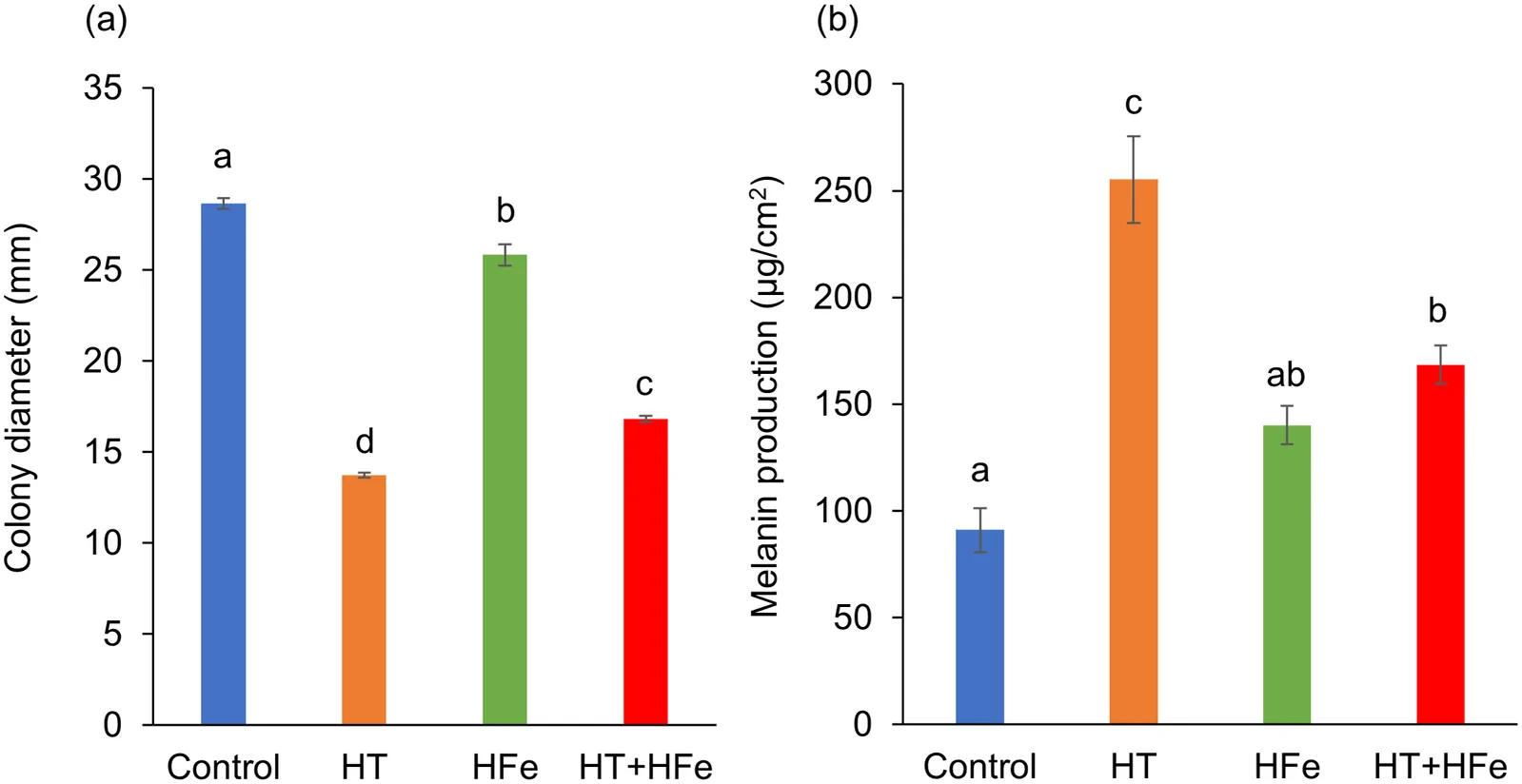
Growth and melanin production of *A. rhizicola* incubated under high Fe and high temperature. *Aquapteridospora rhizicola* was incubated on PDA under control (20°C), high temperature (HT) (30°C), high Fe (HFe) (20°C), and high temperature and high Fe (HT + HFe) (30°C) for three weeks. (a) Colony diameter of *A. rhizicola* and (b) melanin production in *A. rhizicola*. Different letters indicate statistically significant differences among conditions in a one-factor ANOVA, Scheffé’s post-hoc test, p < 0.05 (n = 3). Means are shown with ± SE.

## Discussion

Endophyte-plant symbiosis represents a continuum of interactions ranging from weak pathogenicity to mutualism and is influenced by various factors, including environmental conditions [48]. Our results revealed that the beneficial effects of root endophytes on host plants might not be constitutive, but might be apparent under specific environmental conditions. In the inoculation test using bare soil collected from the sedimentary site under normal temperature conditions, the root-endophytic *A. rhizicola* did not promote the *M. sinensis* seedling growth (Fig 1, Table 1). In contrast, *A. rhizicola* increased the maximum leaf length, FW of terrestrial parts and roots, height, and DW of terrestrial parts under actual soil temperature conditions (Fig 4, Table 4). Several studies have reported that root endophytes isolated from *M. sinensis* promote host-plant growth under metal stress [16,18,28]. *Phialocephala fortinii* increased the root length, FW and DW of terrestrial parts, and root FW of *M. sinensis* in acidic mine soil [28], and increased seedling height in Zn- and Pb-rich soil [18]. Furthermore, *Chaetomium cupreum* promoted root elongation and increased root DW in acidic mine soil [16]. In this study, the effects of *A. rhizicola* on the growth of *M. sinensis* varied between normal temperature and actual soil temperature conditions. While seeds collected in 2020 were used for the normal temperature conditions, due to the depletion of the 2020 seed stock, we needed to use seeds collected in 2022 for the actual soil temperature conditions. Since seeds collected in different years may differ in their physiological states and genetic backgrounds [49–51], a two-tailed unpaired Student’s t-test was separately performed for each condition. Therefore, our results might depend on not only different temperatures but also different physiological states and genetic backgrounds.

IAA produced by root endophytes is known to enhance growth of host plants [52,53]. Our root endophyte, *A. rhizicola*, also produces IAA; therefore, we considered the possibility that *A. rhizicola* would produce IAA under high temperature and high Fe conditions. In the results, although mycelial growth was significantly inhibited compared with the control, IAA concentrations were markedly higher under high Fe (125 ppm), and combined high temperature (30°C) and high Fe (125 ppm) conditions, compared with the control (Fig 7). High temperature (30°C) or heavy metals are also reported to enhance IAA production by endophytes. *Colletotrichum fructicola* isolated from the terrestrial part of *Coffea arabica* L. produced the highest concentration of IAA at 30°C when investigated in the range of 22–37°C [54]. Endophytic *Aspergillus terreus* isolated from salt-tolerant plants, produced the highest IAA at 30°C when investigated in the range of 20–40°C [55]. Additionally, heavy metals (300 ppm each; Zn, Cu, Mn, Cr, or Co) significantly enhanced IAA production by the root endophyte *Mucor* sp. isolated from *Parthenium hysterophorus* L. [56]. *Penicillium funiculosum* isolated from the roots of soybeans (*Glycine max* L.) also increased IAA production under metal stress (2.0 mM of Pb, Cr, Cu, Ni, and Al; total-metal concentration was about 817 ppm via our calculation) [57]. In contrast, apart from our study, no reports have clarified whether endophytes promote IAA production under the combined condition of high temperature and high Fe. This result explains why *A. rhizicola* might enhance *M. sinensis* seedling growth at the sedimentary site under stressful conditions, such as extremely high Fe and sharp temperature fluctuations in bare soil; in other words, *A. rhizicola* might facilitate the establishment of *M. sinensis* at the bare ground of the sedimentary site during the early successional stage.

Endophytes enhance heavy metal tolerance in host plants via various mechanisms. For example, metal sequestration by fungal mycelia from plant cells is an important mechanism for enhancing metal tolerance [56,58]. In fact, root-endophytic *C. cupreum* accumulates Al in its mycelia, which results in the enhancement of Al tolerance in *M. sinensis* at an acidic mine site [16]. Our results showed that the TF of Zn was significantly reduced by *A. rhizicola* inoculation under actual soil temperature conditions compared with the control (without *A. rhizicola*) (Table 5), whereas no significant differences in the TFs of heavy metals were observed under normal temperature conditions between the treatments (Table 2). Given that Zn concentration in the terrestrial parts of *M. sinensis* (> 1000 mg/kg DW) (Fig 5a) exceeded the critical toxic level in plants (100–500 mg/kg DW) [59], and that excess Zn causes oxidative damage to biomolecules [60], *A. rhizicola* might enhance metal tolerance by reducing Zn translocation into the terrestrial parts. This mechanism is consistent with previous studies on DSEs. For example, *Exophiala pisciphila*, isolated from roots of *Arundinella bengalensis* (Spreng.) Druce, inhibits Cd translocation from roots to shoots in maize (*Zea mays* L.) under heavy metal stress (Pb, Zn and Cd) by absorbing metals onto hyphal cell walls [24]. Similarly, *Falciphora oryzae*, isolated from wild rice (*Oryza granulate* L.), reduced the translocation of Cd from roots to shoots in rice grown on solid medium containing high Cd. This reduction was influenced by accumulating Cd in the hyphae and chlamydospores, particularly within vacuoles [58]. Notably, melanin—a major component of the DSE cell wall—possesses heavy metal binding capacity [30,31] and antioxidant activity [32], and possibly helps the host plants tolerate heavy metal stress. In our study imitating the conditions of the bare ground at our study site (high Fe and high temperature), *A. rhizicola* increased the production of melanin 1.9 times higher than that of the control (Fig 8b), although mycelial growth was inhibited 0.6 times compared with the control (Fig 8a). Therefore, *A. rhizicola* might alleviate metal toxicity via melanin production, which may potentially contribute to the suppression of excessive Zn accumulation in the terrestrial parts and enhancement of antioxidant activity through the infection of root cells. Additionally, under high temperature, *A. rhizicola* exhibited the highest production of melanin among all treatments (Fig 8b), although mycelial growth was the lowest (Fig 8a). Few studies have investigated the effect of temperature on melanin production in fungi. *Spissiomyces endophytica* is a DSE isolated from *Balanophora fungosa* JR Forst. & G. Forst and showed the highest melanin production at 25°C when investigated in the range of 15–40°C [61]. Dark-spored *Alternaria brassicicola* is a pathogenic fungus of mustard (*Brassica juncea* (L.) Czern & Coss.), produced the highest concentration of melanin at 25°C when tested between 15, 25, and 35°C [62]. DSEs are also known to protect host plants from high temperature stress; for example, *Curvularia* sp. inoculation enhanced plant biomass of *Dichanthelium lanuginosum* (Elliott) Gould at 45°C under field conditions, because this tolerance might be mediated by cell-wall melanin, which could disperse heat and form a complex with oxygen radicals [63]. Melanin produced by *A. rhizicola* at high temperature might contribute to the adaptation of *M. sinensis* to sharp changes in soil temperature in the bare ground at the sedimentary site.

Notably, root endophytes can also increase host-nutrient uptake [18,26,28,64,65]. Under actual soil temperature conditions, *A. rhizicola* inoculation significantly decreased the Mg concentration in the terrestrial parts of *M. sinensis* while increasing it in the roots (Fig 6); however, under normal temperature conditions, there were no significant differences in nutrient concentrations (Fig 3). In plants, Mg contributes to maintaining membrane stability, promoting compartmentalization of metal ions, and activating antioxidant enzymes to mitigate metal-induced oxidative damage under heavy metal stress [66]. Previous studies have indicated that root-endophytic *P. fortinii* increases the Mg concentration in *M. sinensis* roots, contributing to the enhancement of Al tolerance [28]. As *M. sinensis* accumulates heavy metals in its roots (Fig 5b), the demand for Mg would be higher in the roots than in the terrestrial parts. *Aquapteridospora rhizicola* may effectively enhance heavy metal tolerance of the host by altering the internal distribution of Mg. Several studies have shown that DSEs increase the Mg uptake in herbaceous plants, crops, and trees. For example, DSEs belonging to *Pleosporales* increased Mg concentration in the shoots of *Oryza sativa* L. and *Solanum lycopersicum* L. in pot experiments using non-polluted sand soil [67,68]. Similarly, field studies have shown that DSE colonization of *Galium odoratum* L. roots growing on the beech forest floor displayed a significant positive correlation with Mg concentration in the leaves, thereby suggesting that DSEs could enhance Mg uptake in host plants [69]. In another study, *Vaccinium virgatum* Ait*.* (rabbit eyeberry) infected with DSEs and ericoid mycorrhizal fungi in unsterile peat moss enhanced Mg concentration in the roots compared with the roots grown in sterile peat moss, thereby indicating that DSEs and ericoid mycorrhizal fungi increased Mg uptake in the roots [70]. Similar to these previous studies, *A. rhizicola* promoted Mg uptake in *M. sinensis* roots, which were heavy-metal accumulation sites.

*Miscanthus sinensis* has been reported to grow in heavy metal-rich environments, including mine sites, suggesting that it possesses high heavy metal tolerance by the plant itself [16,18,20,71]. In our study, under normal and actual soil temperature conditions, *M. sinensis* accumulated high concentrations of metals (Al, Cu, Fe and Zn) in the roots (Figs 2b and 5b) compared with general land plants (Al, 40–500 mg/kg DW; Cu, 4–20 mg/kg DW, Fe, 20–700 mg/kg DW; Zn, 1–400 mg/kg DW) [11]. On the contrary, no symptoms of heavy metal toxicity, including growth inhibition, browning of roots, and chlorosis of leaves, were observed in the control and inoculated seedlings (Figs 1 and 4). In this study, we consider visible symptoms to be an important indicator in evaluating toxicity [72,73]. Therefore, it is possible that, under normal and actual soil temperature conditions, *M. sinensis* adapts to high metal accumulation in the roots through its own tolerance mechanisms such as chlorogenic acid production in the roots [18,20] and metal localization in the cell wall (Al; [16]), as shown in previous reports. In addition, endophytes can stimulate the production of host secondary metabolites [74,75]. According to research on *M. sinensis* [34], the root-endophyte *C. cupreum* seemed to increase the secondary metabolite chlorogenic acid, an antioxidant that alleviates heavy metal stress in plants [76], in Al-accumulated roots under hydroponic conditions. In our study, *A. rhizicola* did not increase the chlorogenic acid production in *M. sinensis* roots under normal and actual temperature conditions. Moreover, the concentrations were lower than those in mature *M. sinensis* roots growing at mine sites (0.3–1.2 µg/mg FW of roots; [16,18,20]); therefore, not only chlorogenic acid production but also metal sequestration in the cell wall would be constantly related with metal tolerance in *M. sinensis* itself. Furthermore, the poaceous grass *M. sinensis* is known to be a C_4_ plant [71], which possesses high temperature tolerance [77], suggesting that sharp alteration of temperature in bare ground at our study site is unlikely to impose severe stress on *M. sinensis* itself. Since *M. sinensis* possesses tolerance to metal stress and high soil temperature, the characteristic changes in root-endophytic *A. rhizicola* under high temperature and high Fe might serve as a trigger to establish a symbiotic relationship between the endophyte and *M. sinensis*.

Generally, high concentrations of heavy metals and Al cause a variety of toxic symptoms and are harmful to normal plants [59], and vegetation in some mine sites remains in an early successional stage or in a state of arrested succession even several decades after abandonment [8,78–80]. Pioneer plant species can improve the physical and chemical conditions of mine sites [81,82] and sequentially aid in the further development of vegetation. Therefore, it is important to introduce pioneer plants with heavy metal tolerance for the revegetation of mining sites. *Miscanthus sinensis* is recognized as a pioneer species capable of invading harsh environments such as mine sites [12–15] and acidic sulfate soils in volcanic and coastal regions [83], thereby indicating its high stress tolerance and broad environmental adaptability [71,84]. At our study site, *M. sinensis* patches facilitated the establishment of *P. densiflora* seedlings, which suggests that *M. sinensis* promotes vegetation succession at the mine sites [20]. Our findings showed that the root-endophyte *A. rhizicola* might aid in the establishment of *M. sinensis* at the sedimentary site, thereby suggesting that endophyte-promoted growth could accelerate vegetation succession at the mine site. Furthermore, our results showed that the beneficial effects of root endophytes might be enhanced under native environmental conditions, suggesting that utilizing the symbiotic relationship between locally adapted plants and their native endophytes is advantageous for the ecological revegetation of mine sites. Further studies, including field inoculation tests, are necessary to verify whether *A. rhizicola* contributes to *M. sinensis* establishment and accelerates vegetation development in mine sites.

## Conclusions

Our research indicated that the mutualistic effects of the dark septate endophyte *A. rhizicola* on its host, *M. sinensis*, might be environment-dependent and appear under the harsh environmental conditions native to the sedimentary site, such as high Fe and sharp soil temperature fluctuations. Although *A. rhizicola* did not promote seedling growth in bare soil under normal temperature conditions, this fungus enhanced plant growth under actual soil temperature conditions, possibly via the endophyte’s increased production of IAA under the combined condition of high Fe and high temperature. Furthermore, *A. rhizicola* reduced the translocation of toxic Zn to the host’s terrestrial parts under actual soil temperature conditions, thereby potentially alleviating heavy metal toxicity. This might be explained by the increase of fungal melanin production, which possesses heavy metal binding capacity and antioxidant activity, under the combined condition of high Fe and high temperature. These findings suggest the critical role of native endophyte-plant symbiosis in overcoming barriers to early plant establishment at sedimentary sites. Functional root endophytes isolated from naturally growing plant species at mine sites may enhance greening at mine sites. We hope that locally adapted biological interactions will provide an ecologically advantageous strategy for revegetation and sustainable rehabilitation of degraded mining environments.

## Supporting information

S1 Fig Patches of *M. sinensis* growing at the sedimentary site. *Miscanthus sinensis* naturally grows and forms patches at our study site, which is a sedimentary site in September 2025. *Pinus densiflora* (white arrows) grew inside the *M. sinensis* patch. Scale bar represents 30 cm.
(TIF)

S2 Fig Morphological characteristics of *A. rhizicola* in *M. sinensis* trypan blue-stained roots in both inoculation tests. (a–c) Normal temperature conditions: (a) microsclerotia, (b) endophytic hyphae, and (c) lignitubers. (d–f) Actual soil temperature conditions: (d) microsclerotia, (e) endophytic hyphae, and (f) lignitubers. Scale bars represent 20 µm.
(TIF)

S1 Table Exchangeable cations, available P and Fe, and pH (H_2_O) in non- and γ-ray-sterilized soils. DW: dry weight. Means are shown with ± SE. Differences between non- and γ-ray-sterilized soils were evaluated using a two-tailed unpaired Student’s t-test (n = 4). N.D. indicates that the concentration is below the detection limit.
(PDF)

S2 Table Soil temperatures during inoculation tests under normal and actual soil temperature conditions. Means are shown with ± SE. Differences between the conditions were evaluated using a two-tailed unpaired Student’s t-test (light, n = 2494; dark, n = 1715).
(PDF)

S3 Table Mycelial growth percentage of *A. rhizicola* under different Fe concentrations compared with that of the control. *Aquapteridospora rhizicola* was grown on 1% MA, and then a three-week-old agar disk (5.5 mm i.d.) was transferred onto Czapek agar (2% agar, pH 6.5) or PDA (2% agar, pH 5.0) containing different Fe concentrations (125, 250, 500, and 1000 ppm via addition of FeSO_4_·7H_2_O). As a control, *A. rhizicola* was grown on Czapek agar or PDA in the absence of Fe. The medium was incubated for two weeks at 23°C, and colony diameters at right angles to each other were measured and averaged. Mycelial growth percentage was calculated from the mean diameter (mm) of the colony on the Fe-containing medium and the mean diameter (mm) of the control, which was set to 100%. Means are shown with ± SE (n = 3). – indicates *A. rhizicola* could not grow in this medium.
(PDF)
