# Supplementary material for: Root endophytic Aquapteridospora rhizicola enhances growth and heavy metal tolerance of Miscanthus sinensis Andersson at a sedimentary site via indole-3-acetic acid and melanin production enhanced by high Fe and high temperature
Source: PLoS One. 2026 Oct 27;21(10):e0359285. doi: 10.1371/journal.pone.0359285 (PMC13712714; doi:10.1371/journal.pone.0359285)
Supplement: Supplementary file 1 [file pone.0359285.s003.pdf]

Element (mg/kg DW)	Non-sterilized			Sterilized			P-value
Ca	2621.28	±	60.94	3197.18	±	202.97	0.035
K	N.D.			N.D.			–
Mg	68.84	±	1.71	116.39	±	1.91	< 0.001
Na	N.D.			N.D.			–
P	N.D.			N.D.			–
Fe	170.79	±	16.55	151.76	±	19.98	0.491
pH (H_2O)	7.73	±	0.02	7.76	±	0.02	0.434
