# Supplementary material for: Root endophytic Aquapteridospora rhizicola enhances growth and heavy metal tolerance of Miscanthus sinensis Andersson at a sedimentary site via indole-3-acetic acid and melanin production enhanced by high Fe and high temperature
Source: PLoS One. 2026 Oct 27;21(10):e0359285. doi: 10.1371/journal.pone.0359285 (PMC13712714; doi:10.1371/journal.pone.0359285)
Supplement: Supplementary file 2 [file pone.0359285.s004.pdf]

	Normal temperature conditions	Actual soil temperature conditions	P-value
Light	24.8 ± 0.0°C	31.8 ± 0.0°C	< 0.001
Dark	19.0 ± 0.0°C	21.2 ± 0.0°C	< 0.001
