# Supplementary material for: Root endophytic Aquapteridospora rhizicola enhances growth and heavy metal tolerance of Miscanthus sinensis Andersson at a sedimentary site via indole-3-acetic acid and melanin production enhanced by high Fe and high temperature
Source: PLoS One. 2026 Oct 27;21(10):e0359285. doi: 10.1371/journal.pone.0359285 (PMC13712714; doi:10.1371/journal.pone.0359285)
Supplement: Supplementary file 3 [file pone.0359285.s005.pdf]

Media	Fe concentrations (ppm)			
	125	250	500	1000
Czapek agar	60.4 ± 0.8%	–	–	–
PDA	92.0 ± 1.9%	37.4 ± 2.3%	–	–
